# DNA extract characterization process for microbial detection methods development and validation

**DOI:** 10.1186/1756-0500-5-668

**Published:** 2012-12-03

**Authors:** Nathan D Olson, Jayne B Morrow

**Affiliations:** 1 Biosystems and Biomaterials Division, Material Measurements Laboratory, National Institute of Standard and Technology, 100 Bureau Dr, 20899-8312, Gaithersburg, Maryland

**Keywords:** DNA extraction, DNA characterization, DNA concentration, DNA purity, Shearing, PCR inhibition, qPCR, Biodetection

## Abstract

**Background:**

Quantitative polymerase chain reaction (qPCR) assays used in pathogen detection require rigorous methods development including characterizing DNA extraction products. A DNA extract characterization process is demonstrated using DNA extracted from five different cells types (two Gram-negatives: *Escherichia coli*, and *Burkholderia thailandensis*, spores and vegetative cells from the Gram-positive *Bacillus cereus*, and yeast *Saccharomyces cerevisiae*) with six different methods.

**Results:**

DNA extract quantity (concentration and extraction efficiency) and quality (purity and intactness) varied by cell type and extraction method enabling the demonstration of different DNA characterization methods. DNA purity was measured using UV spectroscopy, where the A_260_/A_280_ and A_260_/A_230_ ratios are indicators of different contaminants. Reproducibility of UV spectroscopy measurements decreased for DNA concentrations less than 17.5 ng/μL. Forty-seven extracts had concentrations greater than 17.5 ng/μL, 25 had A_260_/A_280_ above 2.0, and 28 had A_260_/A_230_ ratios below 1.8 indicating RNA and polysaccharide contamination respectively. Based on a qPCR inhibition assay the contaminants did not inhibit PCR. Extract intactness was evaluated using microfluidic gel electrophoresis. Thirty-five samples had concentrations above the limit of quantification (LOQ, roughly 11 ng/ μL), 93.5% of the DNA was larger than 1kb and 1% was smaller than 300 bp. Extract concentrations ranged from 1502.2 ng/μL to below the LOQ when UV spectroscopy, fluorometry, and qPCR were used. LOQ for UV spectroscopic and fluorometric measurements were 3.5 ng/μL and 0.25 ng/μL respectively. The qPCR LOQ varied by cell type (5.72 × 10^-3^ ng/μL for *E*. *coli*, 2.66 × 10^-3^ ng/μL, for *B*. *cereus*, 3.78 × 10^-3^ ng/μL for *B*. *thailandensis*, and 7.67 × 10^-4^ ng/μL for *S*. *cerevisiae*). A number of samples were below the UV spectroscopy (n = 27), flurometry (n = 15), and qPCR (n = 3) LOQ.

**Conclusion:**

The presented DNA extract characterization process provides measures of DNA quantity and quality applicable to microbial detection methods development and validation studies. Evaluating DNA quality and quantity results in a better understanding of process LOD and contributing factors to suboptimal assay performance. The samples used demonstrated the use of different DNA characterization methods presented but did not encompass the full range of DNA extract characteristics.

## Background

Developments in molecular based microbial detection methods, such as quantitative polymerase chain reaction (qPCR) [1], have significantly contributed to rapid identification and quantification of unknown biological agents [2]. Public health, and clinical laboratories are often tasked with developing qPCR-based assays for detecting unknown pathogens in complex matrices [3,4]. Microbial detection assay development requires optimizing deoxyribonucleic acid (DNA) extraction and qPCR detection assays. DNA extraction is a multi-step process including; lysing the cell wall, isolating the DNA from other cellular materials, and eluting the DNA in a buffer suitable for downstream applications [5]. High sensitivity detection assays require DNA extraction methods with high efficiency, removal of PCR inhibitors, and DNA larger than PCR assay targets [1]. DNA extracts must meet application requirements or assays may result in false negatives with serious consequences [6]. DNA extraction method suitability is determined by characterizing the extracted DNA’s quantity and quality. DNA quantity is an indicator of extraction efficiency and quality parameters (purity and intactness) indicate DNA is free of PCR inhibitors and appropriately sized. DNA quality (purity and intactness) and quantity are evaluated with a number of different techniques.

DNA quality is characterized in terms of purity using UV spectroscopy, presence of inhibitors using a PCR inhibition assay, and intactness using gel electrophoresis. UV spectroscopy is used to evaluate DNA purity by measuring a sample’s absorbance spectrum between 200 and 320 nm, and calculating the A_260_/A_280_ and A_260_/A_230_ ratios [7]. The two-absorbance ratios indicate different contaminants and extract suitability for different applications. For example the A_260_/A_230_ absorbance ratio is a better indicator of suitability for microarrays whereas the A_260_/A_280_ ratio is a better indicator for PCR [8]. Ratios between 1.8 and 2.0 for A_260_/A_280_ are accepted as indicating pure DNA [9]. RNA and protein contamination are indicated by A_260_/A_280_ ratios above and below 1.8 and 2.0 [7,10]. For the A_260_/A_230_ ratio the community accepted range is 1.8 to 2.2 [9], values below this range can indicate phenol, salt, protein or polysaccharide contamination [7,9]. PCR inhibitors, or impurities that interfere with DNA polymerase, lowering PCR efficiency, are assayed for using an inhibition control assay where a known number of exogenous DNA plasmids are added to the extracted DNA and evaluated using qPCR. The presence of PCR inhibitors is identified by an increase in the threshold cycle (C_t_) value for inhibition reactions compared to control reactions. Similarly, DNA fragment size influences detection assay efficiency [11], as efficiency decreases when the qPCR target is fragmented, preventing amplification. Gel electrophoresis is used to evaluate the size distribution of the extracted DNA fragments.

Additional measurement methods are used to evaluate DNA extracts for concentration, and extraction efficiency. DNA quantity is normally measured using three methods: UV spectroscopy, fluorometry, and qPCR. Other methods such as digital PCR and phosphorus elemental analysis are available but not commonly used in diagnostic labs due to specialized equipment requirements, or the amount of DNA required (500 μg for elemental analysis) [12-14]. Two important parameters used to describe quantitative measurement method performance are the limit of detection (LOD, the smallest confidently detected measureable quantity) and the limit of quantitation (LOQ, is the smallest quantity with acceptable repeatability and trueness measurements) [15]. The LOD and LOQ and upper limit of detection vary for UV spectroscopy, flourometry and qPCR. The upper measurement limit is less of a concern compared to the lower limits as the extracts can be diluted to within the measurements working range.

Nucleic acids strongly absorb UV light with wavelengths of 260 nm due to the resonance structure of the purine and pyrimidine bases [7]. The absorbance is converted into ng/μL of double stranded DNA (dsDNA) using the established conversion factor of 50 ng/μL for 1 optical density unit at 260 nm [9]. Other common impurities (including RNA and protein) also absorb at 260 nm, causing the DNA concentration to be overestimated, but do not affect the detection process LOD unless they inhibit the qPCR detection assay. UV absorbance linear range includes absorbance values (A_260 nm_) ranging from 0.1 to 1.0. The LOD and LOQ for UV absorbance measurements are dependent on the instrument and are three and six times, respectively, the standard deviation of ten replicate true blank measurements [15,16].

Fluorescence emission from fluorescently labeled single stranded DNA (ssDNA) or dsDNA is used to estimate DNA concentration with fluorometry measurements [16]. Compared to concentration measurements based on UV spectroscopy, fluorometry is more specific for DNA because fluorescent labels have a higher binding affinity for DNA versus RNA. The limit of detection and quantification for fluorometric DNA concentration measurements is dependent on the fluorescent label and instrument used. A typical limit of detection for 200 μL reactions measured using a plate reader is 2.5 × 10^-4^ ng/μL [17].

Finally, qPCR is applied to measure target sequence copy number concentration. The copy number concentration is equated to DNA concentration based on within genome target sequence copy number and genome mass [18]. qPCR is the most specific method for DNA quantification because it only measures the targeted organism’s DNA, however, qPCR only quantifies intact and accessible targets and does not estimate total DNA. The LOD for qPCR has been reported as 100 copies per reaction [19]. Fewer copies per reaction are detectable but the LOD is dependent on the repeatability of pipetting for low copy numbers. The LOQ is based on the qPCR assay range of linearity.

Several studies have compared DNA extraction methods suitability for microbial detection applications e.g. [20-25], however, valuable information regarding characterization of the DNA extraction products is absent from the extraction method comparison studies. For example DNA extract purity is frequently reported for the A_260_/A_280_ ratio but not the A_260_/A_230_[20-22,25]. Inclusion of the A_260_/A_280_ and A_260_/A_230_ ratio provides additional information about potential PCR inhibitors useful during method optimization. DNA extraction comparison study results are commonly presented in terms of the minimum number of detectable cells, the process LOD, for the different extraction methods and fail to use separate inhibition assays to evaluate extraction method performance. For example in a study by Dauphin et al. [21] the process LOD for DNA extracted using enzymatic lysis with magnetic bead purification was 500 CFUs and other extraction methods evaluated in the study had LODs of 5 CFUs. The difference in LOD could not be attributed to DNA yields as methods with LODs of 5 CFUs had similar yields to the enzymatic lysis and magnetic bead purification method (~ 40 ng). Without results from a PCR inhibition assay there is no direct evidence that inhibitors caused the higher process LOD hindering method optimization. Additionally, independent reports of shearing are infrequently included in DNA extraction comparison studies although shearing can significantly lower the assay efficiency and inclusion would provide valuable information during methods development [26-28].

In order to show the benefits and value in the application of independent methods to characterize DNA extract quality and quantity, DNA was extracted from five cell types using six different extraction methods selected to represent major classes of extraction methods and cell types producing DNA varying in quantity and quality. The extraction methods included a traditional phenol chloroform extraction and five commercial kits utilizing different lysis and purification strategies. The five cell types include two Gram-negatives (*Escherichia coli*, and *Burkholderia thailandensis*), spores and vegetative cells from the Gram-positive *Bacillus cereus*, and the yeast *Saccharomyces cerevisiae*. Extracted DNAs were characterized using multiple methods frequently found in molecular biology laboratories; quantity, characterized by UV spectrometry, fluorometry, and qPCR, quality as defined by spectrometry, independent measures of PCR inhibition and shearing, and the benefits of the different characterization methods are discussed.

## Results

**Figure 1:**
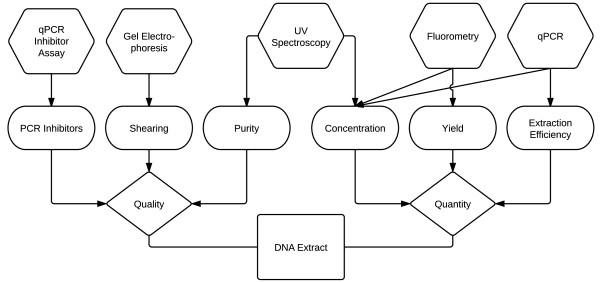
**Flowchart of measurement methods used to evaluate the extracted DNA quantity and quality.** Parameters, characteristics, and measurement methods are indicated in diamond, oval, and hexagon blocks, respectively

DNA extracted from five different cell types using six different extraction methods (Table 1) was applied to DNA characterization methods as shown in Figure 1. DNA extract analysis is presented in terms of quality and quantity.

**Table 1:** Extraction methods by application and processes

**Method**^**a**^	**Intended sample type**	**Lysis method**	**Purification method**
Reference	General	Mechanical - Bead Beat	Phenol Chloroform
PrecipB	Biofilm	Mechanical - Bead Beat	Precipitation and Silica Column
MagBeads	General	Mechanical - Bead Beat	Magnetic Beads
PrecipS	Soil	Mechanical - Bead Beat	Precipitation and Silica Column
ChemLysis	Gram Negative	Chemical - Enzymatic	Silica Column
PrecipG	General	Mechanical - Bead Beat	Precipitation and Silica Column

^a^ DNA extraction kits are represented based on the fundamental methods utilized by each kit rather than kit names. Kits included MoBio PowerBiofilm (precipB), MoBio PowerSoil (precipS), MoBio UltraClean (precipG), Qiagen DNAeasy with Gram Negative lysis protocol (chemLysis), and Idaho Technologies Platinum Path (magBeads). Certain commercial equipment, instruments, or materials are identified in this paper in order to specify the experimental procedure adequately. Such identification is not intended to imply recommendations or endorsement by NIST, nor is it intended to imply that the materials or equipment identified are necessarily the best available for the purpose.

### DNA quality

Extract quality was reported in terms of purity by UV spectroscopy, PCR inhibition using an independent qPCR assay, and intactness using gel electrophoresis.

**Figure 2:**
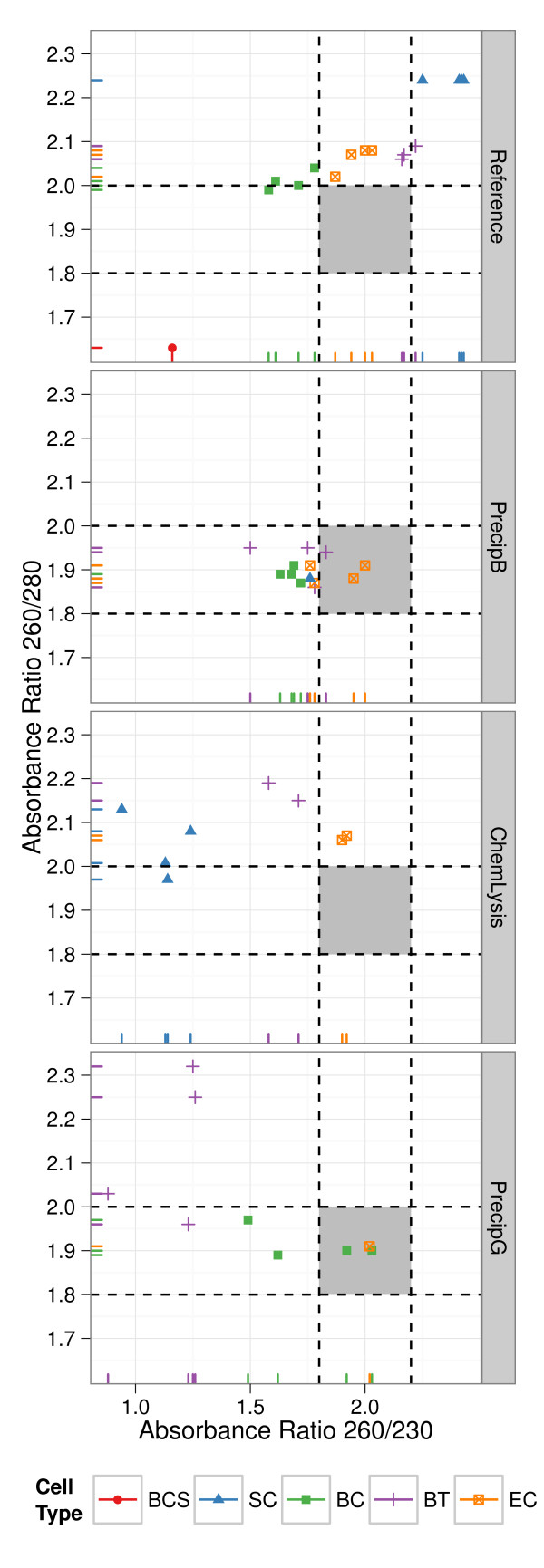
**Scatter plot DNA extract purity (A**_**260**_**/A**_**280 **_**nm *vs. *A**_**260**_**/A**_**230 **_**nm).** Only samples with DNA concentrations greater than 17.5 ng/μL were included in the analysis. Graphs are separated by extraction method. Cell types are indicated by the data point shape, color, and rug plot color: *B. cereus* spores (n = 1 extracts), Red ●, *S. cerevisiae* (n = 9 extracts), blue ▲, *B. cereus* vegetative (n = 12 extracts), green ■, *B. thailandensis* (n = 15 extracts), purple +, and *E. coli* (n = 11 extracts)*,* yellow ⊠. Dotted lines indicate the upper limits of community accepted purity ratios; light grey areas show the lower limits of community accepted purity ratios

UV absorbance ratios, A_280_/A_260_ and A_230_/A_260_, were used to evaluate DNA extract purity (Additional file 1: Table S1). Nanodrop-1000 performance was assessed with a dilution series of a control DNA sample (Human DNA Quantification standard SRM 2372 part A, Additional file 2: Figure S1). The reproducibility of the ratio values decreased for samples with measured concentrations less than 17.5 ng/μL (Additional file 2: Figure S1). Of the 108 DNA extracts examined, 47 had concentrations greater than 17.5 ng/μL. All but one of the extracts (a *B*. *cereus* spore sample extracted using the reference method) had A_260/_A_280_ ratios above 1.8. Extraction methods with a precipitation step as part of purification had the highest proportion of extracts with purity ratios within the accepted range of 1.8 to 2.0 for the A_260_/A_280_ ratio (grey box in Figure 2). A number of extracts had A_260_/A_280_ ratios above 2.0. A high proportion of samples extracted using the reference (14/17) and chemLysis (7/8) methods as well as *S*. *cerevisiae* (7/9) extracts had A_280_/A_260_ ratios above 2.0 (Figure 2).

A_260_/A_230_ ratio, an indicator of RNA, phenol, salt, protein, and polysaccharide contamination, are presented in Figure 2. Most of the extracts (28 out of 47) had A_260_/A_230_ ratios below 1.8, and 6 extracts had ratios above the accepted range (grey box in Figure 2). All extraction methods had low proportions of extracts within the accepted ratio. The reference method and precipB had purity ratios within or near the accepted ratios, 2.00 ± 0.34 and 1.75 ± 0.13 (mean and standard deviation) respectively. A high proportion *E*. *coli* extracts, 9 out of 11, were within the accepted ratio. Of the 6 extracts with A_260_/A_230_ ratios above 2.0 four were from *S*. *cerevisiae*.

**Figure 3:**
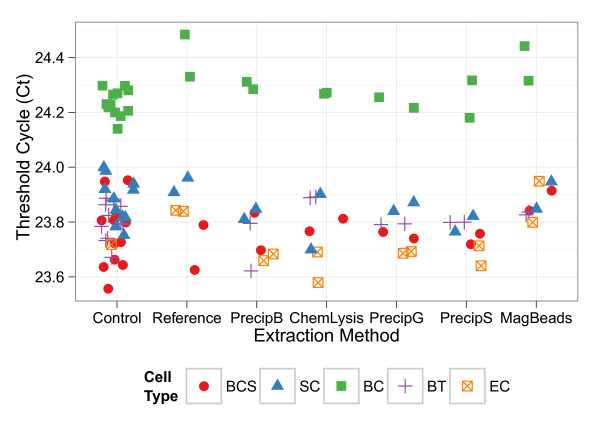
**Evaluation of PCR inhibition for extracts from five cell types.** Data points represent the median cycle threshold (Ct) values for n = 3 replicates. Shape and color indicate the source cell type, *B. cereus* (spore), red ●, *S. cerevisiae* , blue ▲, *B. cereus*, green ■, *B. thailandensis*, purple +, *E. coli*, orange ⊠, of the extracted DNA, respectively

Extracted DNA was assayed for PCR inhibitors using a detection independent inhibition assay, where a known number of exogenous DNA plasmids were added to the extracted DNA and evaluated using qPCR. The range in C_t_’s (threshold cycle) for each individual 96 well plate was less than 0.4 cycles, and the difference between the mean control C_t_ for a plate and the inhibition reactions was less than 0.26 cycles (Figure 3). For inhibited samples the expected difference in C_t_ values between the control and inhibition reactions is greater than 1 cycle, therefore no inhibition was observed. The mean C_t_ values for the *B*. *cereus* vegetative cells 96 well plate was 0.48 cycles greater than the mean for all other plates, this observed difference is due to run to run variation and not inhibition (Figure 3).

Finally, DNA shearing was evaluated using microfluidic gel electrophoresis. A cutoff value for quantitative analysis was set at 100 fragment concentration (FC), roughly 11 ng/μL of DNA, as fluorescent reads below this cutoff were near the limit of detection preventing accurate size percentage calculations. Thirty-five samples had FC values above the cutoff with 93.5% (65.7% - 100%, mean and percent range) of DNA larger than 1 kb and 1.0% (< 1% - 10.0%) less than 300 bp in size (Additional file 3: Figure S2). Slight shearing was observed for some of the vegetative cell samples extracted with methods using bead beating during the lysis step (Additional file 3: Figure S2). For example one *B*. *thailandensis* and one *B*. *cereus* sample extracted using precipB had broad peaks ranging in size from 450 bp to over 17 kb with 77.5% and 81.1% of the DNA larger than 1 kb respectively (Additional file 3: Figure S2, precipB row). For extracts from vegetative cells the reference method, which included a bead beating lysis step, produced fragments less than 300 bp in size comprising 1.7% (0% - 10.0%, mean and range) of the extracted DNA (Additional file 3: Figure S2, Reference row).

### DNA quantity

LOQs were established for the three different DNA concentration methods used to assess the quantity of DNA in extracts. LOQ for the Nanodrop based DNA measurements was set at 3.5 ng/μL; 6 times the standard deviation of 10 true negatives [15]. In comparison, the Qubit high sensitivity dsDNA LOQ was set at 0.25 ng/μL for this study, as the variability of replicate measurements increased for dilutions with measured concentrations below this value (Additional file 4: Figure S3). The LOQ for qPCR varies by assay and the LOQ is dependent on the qPCR efficiency, the number of copies of the target sequence within the organism’s genome, and the organism’s genome size. The qPCR LOQ for the assays in this study were; 5.72 × 10^-3^ ng/μL for the *E*. *coli*, 2.66 × 10^-3^ ng/μL, for *B*. *cereus*, 3.78 × 10^-3^ ng/μL for *B*. *thailandensis*, and 7.67 × 10^-4^ ng/μL for *S*. *cerevisiae*. The actual copy number LOQ for the three assays ranged from 1000 to 248 copies per reaction. A number of samples were below the limit of quantification of the Nanodrop (n = 27) and Qubit HS assay (n = 15). Three of the 108 samples were below the qPCR assays LOQ.

Overall DNA concentration ranged from 1502.2 ng/μL to below the LOQ, depending on characterization and extraction methods (Table 2). The DNA concentration measurements made using the Nanodrop were statistically greater than the other two (for example measurement methods for the reference (p < 0.00), chemLysis (p < 0.00), and precipS (p < 0.03), (Table 2).

**Table 2:** DNA concentration ng/μL, presented as median (maximum – minimum) measured using three different measurement methods

**Extraction method**	**UV spectroscopy**	**qPCR**	**Fluorometry**
Reference	524.4 (1502.2 − <LOQ^*^)	144.2 (487.7 − 0.02)	84.2 (460.0 − <LOQ)
PrecipB	61.1 (178.3 − 3.8)	101.5 (149.4 − 0.00)	51.8 (178.4 − 3.8)
ChemLysis	16.5 (110.2 − <LOQ)	2.1 (47.7 − 0.19)	2.2 (55.2 − <LOQ)
PrecipG	14.9 (44.4 − <LOQ)	8.4 (94.5 − 0.17)	6.2 (29.6 − <LOQ)
PrecipS	4.0 (12.0 − <LOQ)	1.5 (6.1 − 0.01)	1.4 (9.5 − <LOQ)
MagBeads	4.3 (7.6 − <LOQ)	0.2 (1.3 − <LOQ)	0.4 (2.4 − <LOQ)

^*^ below the method limit of quantification (LOQ).

**Figure 4:**
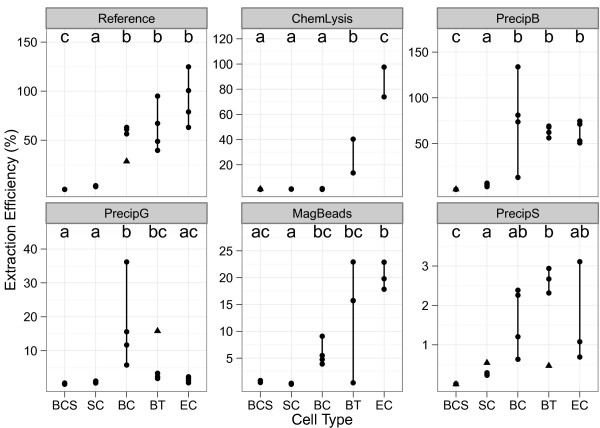
**DNA extraction efficiency as a function of cell type quantified by qPCR.** Cell types are labeled as follows: *B. cereus* (spore), BCS, *S. cerevisiae*, SC, *B. cereus*, BC, *B. thailandensis*, BT, *E. coli*, EC. Vertical lines indicate the dispersion of the data; replicate outliers are indicated as ▲ data points. Black letters (a, b, c) indicate statistical differences within each plot, based on a Tukey’s HSD test with p < 0.05, when the outliers were excluded in the analysis. Plots are grouped by extraction method. Scales are independent for each graph due to the large range in responses

Calculation of extraction efficiencies using qPCR concentration values allow for a more direct comparison with the literature where it is common to present extraction methods in terms of the overall process efficiency. Extraction efficiencies varied significantly (p < 0.05) by both cell type and extraction method, with efficiencies ranging from less than 0.0001% to over 100% (Figure 4). Extraction efficiencies in general were higher for the two Gram-negative organisms than the hard to lyse cells types; yeast, spores, and Gram-positive. For example for extractions using chemical lysis the extraction efficiency for the hard to lyse cell types was less than 1% and statistically lower (p < 0.05) than the Gram-negative, *B*. *thailandensis* and *E*. *coli* with mean extraction efficiencies of 27% and 86% respectively. The three precip extraction methods all used bead beating for lysis with precipitation and silica spin columns for purification but had different ranges in extraction efficiency (Figure 4C,D,F).

## Discussion

The purpose of this work was to evaluate commonly used DNA characterization methods for applicability to inform the assay developer of DNA extraction method performance. The results are discussed in terms of DNA extract quality and quantity.

### DNA quality

DNA extract purity is of interests in terms of how contaminants will affect downstream assay performance. UV spectroscopy provides an indicator for different types of extract contaminants such as proteins, polysaccharides, and RNA [7]. PCR inhibition assays indicate whether contaminants will adversely affect the qPCR detection assay [29]. Purity ratios, obtained using UV spectroscopy, were outside the accepted range (1.8 to 2.0 for A_260_/A_280_ and 1.8 to 2.2 for A_260_/A_230_) for most extracts but no PCR inhibition was observed.

High A_260_/A_280_ absorbance ratios, indicating RNA co-extraction [30,31], were observed for 25 of the 47 samples with sufficient DNA concentration for reliable purity ratio measurement. The reference method had the highest proportion of extracts with A_260_/A_280_ ratios above 2.0, most likely due to RNA co-extraction with acidic phenol (phenol chloroform had pH of 5.2) [5]. A high proportion of the *S*. *cerevisiae* extracts also had high A_260_/A_280_, the reason for the suspected RNA contamination is unknown but possibly due to the difference in mRNA decay between prokaryotes and eukaryotes [32]. RNA in DNA extracts does not interfere with downstream applications but does cause overestimation of UV spectroscopy determined DNA concentrations [7].

Humic acids, proteins and polysaccharides, indicated by low A_260_/A_230,_ adversely affect PCR amplification kinetics [7,8,33,34]. A majority of the extracts in this study had low A_260_/A_230_ purity ratios. The contaminants are likely polysaccharides as humic acids are found in soil and sediment environmental samples and not associated with cells [35,36] and protein contamination would have caused low A_260_/A_280_ ratios which were not observed [7]. Extraction methods are often optimized to enhance the removal of polysaccharides for example including the use of the surfactant ctyl trimethylammonium bromide or precipitation with high salt concentrations [36,37]. Similarly, the precipitation purification step included in three of the extraction methods reduced DNA contaminants as indicated by the higher proportion of extracts with purity ratios within the accepted range compared to the other extraction methods (Figure 2). For detection of pathogens in samples that are rich in polysaccharides it is essential to optimize the extraction method for their removal [30,37].

The primary limitation to UV spectroscopic DNA purity measurements is that the measurement only provides indicators for different types of contaminants and no information about the effect of these contaminants on downstream applications. Additionally, purity assays are sometimes used when specific contaminants are of interest, such as measuring UV absorbance of a sample at 320 nm for humic acid detection [38]. A second limitation to the method is the required sample concentration, samples with measured concentrations less than 17.5 ng/μL were found to have unreliable purity ratios and purity measurements were only available for half of the extracts characterized in the study.

Few reports explore the link between extract quality and PCR assay performance and/or optimization. PCR inhibitors are often associated with the sample matrix, including blood, food, water and soil [36]. For example, polysaccharides are known to interfere with downstream detection of plant diseases and pathogen contamination in food and water [36]. Although no inhibitors were expected because pure cultures were applied in this study, it is important to run inhibition assays to ensure the extracted purity is suitable for downstream applications. PCR inhibitors can lead to false negatives or underestimation of the quantity of a biological agent. qPCR assay susceptibility to inhibition varies by polymerase, primer regions, and target sequence [39-41]. Even though two different assays, one for inhibition and one for DNA quantification by qPCR, were used in this study and no inhibition was observed it is not likely that inhibitors were not detected as Qubit and qPCR measurements were in agreement (Table 2).

DNA fragmentation can decrease qPCR efficiency or cause the reaction to fail completely [28,42] resulting in higher process LODs. Assays requiring larger target sequences are more likely adversely affected by shearing [27,28]. When DNA extracts are evaluated for shearing it is commonly performed using standard agarose gel electrophoresis [26,43,44]. The use of microfluidic gel electrophoresis allows for a lower limit of detection (11 ng per sample compared to 20 ng per band) and semi-quantitative shearing analysis compared to standard gel electrophoresis [45]. The ideal target length for qPCR is 50 bp-200 bp [46]. Overall 1.0% (0% - 10.0%, mean and range) of the extracted DNA was less than 300 bp and therefore the degree of shearing observed for the DNA extracts did not impact qPCR performance. Slight shearing was observed for a number of the vegetative cell samples extracted with the bead beating method. Bead beating causes shearing but as observed in this study the degree of shearing varies and does not adversely affect most PCR applications using large quantities of genomic DNA [43,47].

### DNA quantity

Accurate DNA quantity measures are critical to assay optimization as losses in DNA due to extraction procedures contribute to a reduction in overall detection assay performance [25,38]. While more accurate DNA concentration measurement methods are available including digital PCR and phosphorus elemental analysis [12-14] the methods used in this study represent those that are commonly used in molecular and diagnostic laboratories. The three DNA concentration measurement methods used in this study were UV spectroscopy, spectrofluorometry, and qPCR.

Each of the concentration methods used has different assumptions, and limitations. For example UV spectroscopy based concentration measurements requires two assumptions. First, DNA is the only molecule in the extract that absorbs light at 260 nm and second, the DNA is all double-stranded. The UV spectroscopy DNA concentration measurements were statistically higher than the other two measurement methods (Table 2). The A_260_/A_280_ and A_260_/A_230_ absorbance ratios above the acceptable range observed for a number of extracts indicated that co-extracted RNA caused the A_260_ measurement to overestimate DNA concentration (Table 2). Absorbance based DNA concentration measurements commonly overestimate DNA concentrations for environmental samples due to contaminants from the sample matrix [48]. Accuracy of absorbance based DNA concentration measurements is dependent on sample purity [48,49]. For extracts known to contain contaminants such as RNA and protein other DNA concentration measurement methods such as spectrofluorometry and qPCR are more suitable than UV spectroscopy. The primary advantage of UV spectroscopy is the availability of microvolume instruments that are faster, easy to use, and requires less sample volume [48].

The Qubit and other bench top fluorometers allow for relatively fast DNA concentration measurements that are not as adversely affected by DNA contaminants as UV spectroscopy [45]. The concentration measurements for qPCR and Qubit were in agreement (Table 2). A number of DNA extracts (n = 15) were below the identified method LOQ of 0.25 ng/μL and therefore the measurement method was not suitable for hard to lyse cells that had extracts with low concentration (Table 2, Additional file 4: Figure S3). Fluorometric DNA concentration measurements do not measure ssDNA in the sample and can lead to DNA concentration underestimation [45]. The accuracy of the fluorometric DNA concentration measurements is dependent on the accuracy of the DNA standards used [19]. Ideally the DNA concentration standard used should have a certified concentration that is traceable to the SI and has a published uncertainty value [19]. The standards in DNA concentration assay kits do not always meet this requirement and the concentration measurements should be evaluated with this in mind [50]. The advantage of using fluorometric DNA concentration measurement methods compared to qPCR is that no additional assay development is required for individual organisms.

Quantitative PCR (qPCR) was the third method used to measure DNA concentration. DNA concentration measurements made using qPCR assume the number of within genome DNA sequence target copies is known and constant and that whole genomes are extracted [18]. qPCR had the lowest observed limit of quantification (~10^-3^ ng/μL depending on the assay) and the fewest number of samples with concentrations below the method LOQ (n = 3). The primary limitation to using qPCR for measuring DNA concentration is that; method development, validation, and execution are significantly more time intensive and costly than either of the two other methods. However, qPCR is the only measurement method that measures the DNA concentration of a specific organism in a mixed sample (e.g. detection of pathogens in food or tissue samples). As with fluorometric DNA concentration measurements qPCR concentration measurements require the use of a standard. The standard used can be a major source of qPCR measurement uncertainty [19]. No standard reference materials are available for the qPCR assays used in this study and the associated uncertainty of the standards used in this study was unknown.

Finally, extraction efficiencies ranged by method and even varied for the three methods that used precipitation and silica spin columns for purification and bead beating for lysis indicating that extraction efficiency was dependent on more than the fundamental properties of the extraction methods. It is important to note when discussing reported extraction efficiencies that quantity and quality are factors of the kits intended use as there is a tradeoff between the two. For example the magBeads extraction method was designed for use in field applications with limited resources and user experience. While the concentration of the resulting DNA was low, the extraction efficiency was comparable to the other extraction methods (Table 3, Figure 4). Similarly, the lysis step for the chemLysis method was optimized for Gram-negative bacteria as evident with the higher extraction efficiency for *B*. *thailandensis* and *E*. *coli* compared to the other cell types. Soils are rich in PCR inhibiting humic acids [36] and the precipS extraction method, optimized for extracting DNA from soils, included a purification step to remove humic acids. Additional, steps may have resulted in loss of genomic DNA. A range of different size beads are used in the bead beating step of precipB which may aide in lysis of different cell types resulting in the statistically similar extraction efficiencies between all vegetative cell types not observed for the other extraction methods (Figure 4). Analysis of DNA yields using different size beads found that higher yields for hard to lyse *Mycobacteria* were obtained using smaller sized beads (0.1 mm vs 0.5 mm), but no studies have empirically evaluated lysis efficiency for different cell types using different size beads [51]. For precipG quantity was sacrificed as the quantity of the intended sample type, microbial cultures, is not limited.

**Table 3:** DNA primers used in quantity qPCR assays

**Organism**	**Gene target (amplicon size)**^**a**^	**Name**	**Sequence**	**Con**^**b**^	**AE**^**c**^	**Ref.**
*E*. *coli*	*lacZ* (70)	lacZF1	CCT GAG GCC GAT ACT GTC GT	3	0.99	[52]
		lacZR1	TTG GTG TAG ATG GGC GCA T	3		
*B*. *thailandensis*	*fliC* (62)	fliCF1	AGC AGA TCT CGG AAG TGA ACC	2	0.92	This study
		fliCR1	GAG GAT GTT CTT GCC GTT GT	2		
*B*. *cereus*	*pc*-*plc* (144)	PCER-F	GGA TTC ATG GAG CGG CAG TA	3	0.95	[53]
		PCER-3R	GCT TAC CTG TCA TTG GTG TAA CTT CA	2		
*S*. *cerevisiae*	26S^d^ (124)	YEAST-F	GAG TCG AGT TGT TTG GGA ATG C	3	0.91	[54]
		YEST-R	TCT CTT TCC AAA GTT CTT TTC ATC TTT	2		

^a^ Amplicon size in base pairs.

^b^ Optimized primer concentration (μM).

^c^ Amplification Efficiency, calculated using the slope of the standard curve.

^d^ Assay targeted the D1/D2 variable region of 26S.

## Conclusions

During optimization of detection assays and determining which DNA extraction method to use, downstream application requirements and common sources of downstream application inhibitors [55-57] will dictate what methods are chosen to characterize DNA extract quality and quantity. When optimizing for true unknowns it is advantageous to use extraction methods that are optimal for hard-to-lyse cells. The limited number of cell types and extraction methods evaluated here were not intended to be exhaustive or to guide extraction method selection but rather to present the limitations and advantages of different extract characterization methods. The use of only a single sample type was a limitation of the study and additional sample types would have better challenged the inhibition assay. Along the same lines the use of more intense mechanical lysis procedures such as longer bead beating steps or sonication may have produced DNA extracts that better challenged the shearing characterization assay. More importantly, the study provides a procedural model for assessing DNA extract quantity and quality that can be applied when evaluating extracts for other microbiology fields such as microbial ecology marker gene studies and shotgun metagenomics. The extract characterization methods presented here can serve as a starting point for the development of a standard procedure for evaluating DNA extract quality and quantity for universal applications in the study of microbial systems.

## Methods

### Culture preparation and cell enumeration

DNA was extracted from five cell types: the Gram negative *Burkholderia thailandensis* (ATCC700388) and *Escherichia coli* 0157:H7 non-toxic strain (ATCC700728), Gram positive *Bacillus cereus* vegetative cells and spores (ATCC10987), and the eukaryote *Saccharomyces cerevisiae* (ATCC 204516). Vegetative cell types were cultured in 20 mL of Luria-Bertani (LB) broth with shaking (30 rpm) at 30°C overnight. *S*. *cerevisiae* was grown in potato dextrose broth overnight at 37°C with shaking. Cultures were washed and stored in 1 mL aliquots at – 80°C until extraction or enumeration. Aliquots were stored in either phosphate buffered saline with 0.04% Tween 80 (PBST) or PBST with 10% glycerol (PBST-gly). *B*. *cereus* spores were prepared using the procedure described in Da Silva et al. [58], using PGSM sporulation media and stored in 47.5% ethanol at 4°C. Sporulation media was comprised of 1.5% agar, 7.5 g nutrient broth (Difco Bacto-peptone; VGD, Inc.), 1 g glucose, 3.4 g KH_2_PO_4_, and 4.35 g K_2_HPO_4_ in 1 L sterile deionized water. After the media was autoclaved, 5 mL of filter sterilized CaCl_2_ (0.0366 g/mL) and a salt solution (0.0246 g/mL MgSO_4_, 4 × 10 ^-4^ g/mL MnSO_4_, 0.0028 ZnSO_4_, 0.004 g/mL FeSO_4_) was added.

All cultures were enumerated using a Petroff-Hausser Counting Chamber (Huasser Scientific, Horsham, PA, USA). Samples were diluted 10 fold to a countable concentration in PBST, and triplicate samples were visualized and enumerated using phase contrast microscopy with an Olympus BX51 microscope.

### DNA extraction

DNA was extracted from culture preparations (1–5 × 10^9^ cells/mL) using six extraction methods utilizing different lysis and purification procedures (Table 1). Extraction methods were chosen based on their ability to produce DNA extracts with a range of quantity and quality characteristics that represented general classes of lysis and purification methods. Four extractions were performed for all cell types except for *B*. *cereus* spores, which were extracted in triplicate for each of the extraction methods. Due to limited sample size only two extractions were performed using the chemLysis method for *E*. *coli* and *B*. *thailandensis*, and three *E*. *coli* samples were extracted with precipS. The manufactures’ protocols for all commercial extraction methods were followed excluding using the specified sample type for precipS and precipB methods and no RNAase step was performed for the chemLysis extraction method. For the chemLysis extraction method the manufacture’s Gram-negative lysis protocol was used. Bead beating steps were performed using the Vortex-Genie 2 (MoBio Laboratories, Inc. California, USA) set at the maximum speed with a MoBio vortex adapter. Extracted DNA was stored at −20°C until DNA quantity and quality analysis.

A non-commercial phenol-chloroform based protocol was used as a reference method [9]. Culture aliquots were pelleted by centrifugation (13,000 × *g* for 3 min) and resuspended in 500 μL 1 × TE buffer (Tris-EDTA, pH 8, Fisher Bioreagents, New Jersey, USA). The re-suspended pellet was transferred to a 2 mL tube with screw caps (BioSpec Products, Inc. Oklahoma, USA) with 0.8 g zirconia/silica beads (BioSpec Products, Inc.). Tubes were bead beaten for 20 min then centrifuged for 30 sec at 10,000 × *g*. The supernatant was transferred to a new tube to which 800 μL phenol chloroform 1:1 (MP Biochemicals, Ohio, USA) was added then vortexed for 30 s to mix. Tubes were centrifuged at 10,000 × *g* for 10 min and the aqueous phase was transferred to a new tube. Next, 500 μL chloroform isoamyl alcohol 24:1 (Acros Organics, New Jersey, USA) was added and the solution was vortexed again for 30 s. Aqueous and polar phases were separated by centrifugation (10,000 × *g* for 10 min). Aqueous phase was transferred to a new tube to which 1 mL of absolute ethanol and 50 μL of 3M sodium acetate was added. Tubes were vortexed briefly then incubated at −20°C for 30 min. DNA was pelleted by centrifugation (13,000 × *g*, 10 min) and washed with 70% ethanol. The washed pellet was allowed to air dry for 30 min then re-suspended in 50 μL of 0.1 × TE and stored at −20°C.

### DNA quality

DNA extract quality was reported based on independent measurements of purity and PCR inhibition DNA (Figure 1). The DNA UV absorbance measured using a Nanodrop-ND1000 (Thermo Fisher Scientific, Waltham, MA) provides an assessment of contaminants (polysaccharides and proteins). The absorbance ratios for A_280_/A_260_ and A_260_/A_230_ were determined for 2 μL samples using ND-1000 V3.8.1 software (Thermo Fisher Scientific, Waltham, MA). Additionally, a dilution series of a control DNA (Human DNA quantification standard SRM 2372 part A, National Institute of Standards and Technology, Gaithersburg, MD) was used to assess the precision of the purity ratio measurements for different DNA concentrations. The SRM 2372 at the time of certification was double stranded. Overtime an unknown proportion of the DNA standard has become single stranded. Due to the change in strandedness of the DNA standard the certified UV absorbance values are longer representative of the standard and cannot be used in assessing instrument performance and measurement accuracy.

#### PCR inhibition assay

The presence of PCR inhibitors in DNA extracts was determined using an inhibition control assay. Reactions were run on the ABI 7900 HT Real Time PCR System following manufacturers recommended thermocycling profile. Inhibition reactions of 20 μL included 1 × TaqMan® Environmental Master Mix 2.0 (Life Technologies, Grand Island, NY), 1× ERCC-00095 assay (Ac03459926_a1, Life Technologies, Grand Island, NY), 750 copies of ERCC-00095 plasmid (SRM 2374 candidate material with a certified DNA sequence; National Institute of Standards and Technology, Gaithersburg, MD), and 2 μL of extracted DNA being assayed for PCR inhibitors. Duplicate extractions for each cell type and extraction method combination were evaluated for the presence of inhibitors; excluding *B*. *thailandensis* reference method extractions, due to limited DNA. Cell types were run independently on separate 96 well. Twelve plasmid controls reactions, where no extracted DNA was added, were included in each run, excluding the *E*. *coli* cell type, to evaluate within and between run variability. The SDS v2.4 software (Life Technologies, Grand Island, NY) with default settings was used to calculate the threshold cycle (C_t_).

#### DNA shearing

DNA shearing was evaluated by microfluidic gel electrophoresis with the Agilent Bioanalyzer 2100 and DNA 12000 assay (Agilent Technologies, Inc. Santa Clara, CA) according to manufacturer’s instructions. The Agilent Bioanalyzer measures fluorescence intensity emitted by fluorescently labeled dsDNA as it passes through the detector and results are electropherogram plots. Time to detect and fluorescence are proportional to DNA size and concentration. The total amount of DNA detected, the percentage of DNA > 300 bp and < 1 kb was calculated in R (version 2.15.0) as the area under the electropherogram using the trapezoid method (ROC package), with area under the curve (FU × s), presented as fragment concentration (FC).

### DNA quantity

#### DNA concentration limit of detection and quantification

DNA quantity was measured using three different methods; UV spectroscopy, spectrofluometry, and quantitative PCR (qPCR) (Figure 1). The UV spectrophotometer, Nanodrop-ND1000 (Thermo Fisher Scientific, Waltham, MA), was used to measure DNA concentration, wherein 1 optical density at 260 nm is equivalent to 50 ng/μL of dsDNA [9]. The limit of quantification for Nanodrop measurements was defined as 6 times the standard deviation of 10 true negative replicates [15]. Spectrofluorometric DNA concentration measurements were made using Qubit (Invitrogen, Carlsbad, CA). Qubit assays were performed following the manufacture’s protocol. The Qubit broad range assays was used to measure DNA concentration, for samples with concentrations less than 1 ng/μL the Qubit high sensitivity assay was used. The standard deviation of true negatives principle used to determine the LOQ for the Nanodrop is not applicable to Qubit measurements as DNA concentrations out of range of the standard curve are stated as such by the instrument and therefore no values are provided for true negatives [15]. The LOQ for the Qubit high sensitivity assay was determined using a dilution series of a control DNA (Human DNA Quantitation Standard SRM 2372 part A). Genomic DNA copy number was determined using organism specific qPCR assays (Table 3). qPCR LOQ (*qLOQ*, ng/μL) values were determined by first calculating the genome mass (*GM*, ng/ genome) based on the size of the organism’s genome (*GS*, bp/ genome) and the established mass to bp constant of 0.978 × 10^12^ bp/ng (Eq. 1) [18,19,52].

(1) $$\;GM=\frac{GS}{0.978\times 10^{12}}$$

*qLOQ* (ng/μL) values were then determined based on the plasmid copy number for the lowest dilution in the standard curve (*pLD*, copies/μL) the number of target sequences per genome (*CNg*, copies/ genome), and the mass of the organisms genome (*GM*, ng/ genome) which was calculated using equation 2.

(2) $$\;qLOQ=\frac{\left(pLD\times GM\right)}{CNg}$$

#### qPCR concentration assays

For the qPCR assays, primer sequences were obtained from the literature except the sequences obtained for the *Burkholderia thailandensis* assay, which were designed using Primer3Plus (http://www.bioinformatics.nl) to target the *fliC* gene, which was used in previous studies to quantify *Burkholderia* species [59]. Primer specificity was verified *in*-*silico* using Primer-BLAST (http://www.blast.ncbi.nlm.nih.gov). Standard curves of plasmids containing PCR amplified target sequences were used for absolute quantification. Plasmids containing PCR target sequences were produced using the pGEM-T Easy Vector System (Promega Corp., Madison, WI) according to manufacturer’s protocol, purified using the QIAprep Spin Miniprep Kit (Qiagen Inc., Valencia, CA), and linearized by Fast Digest SacI restriction digest (Fermentas Inc., Glen Bernie, MD). Plasmid DNA concentration was determined using the Qubit Broad Range Assay.

qPCR reactions were run on the 7900 HT Real Time PCR System (Life Technologies, Grand Island, NY). Samples, standard curves, and no template controls were run in triplicate with 1× Power SYBR® Green PCR Master Mix (Life Technologies, Grand Island, NY), primers at optimized concentrations, and molecular grade nuclease-free water (Life Technologies, Grand Island, NY) was added for a final volume of 20 μL. Between 1 ng and 10 ng of extracted DNA was added to sample reactions, molecular grade water was added to the no template control reactions in place of extracted DNA, and a 10 fold plasmid dilution series was added to standard curve reactions. Primer concentrations were optimized according to Nolan et al. [46]. Reaction specificity was verified using melt curve analysis, and r^2^ values were determined using SDS v2.4 software (Life Technologies, Grand Island, NY) with default settings. *B*. *cereus* and *S*. *cerevisiae* samples were run on two 96 well plates, inter-run calibrators (IRCs) were used to normalize for run-to-run variation [60].

#### qPCR concentration values

qPCR DNA concentration was calculated first by determining the concentration of genome equivalents (c*GE*, genome equivalents(*GE*)/μL, Eq. 3) in the extract based on the median of triplicate target sequence copy number concentrations in the extract determined using qPCR (*qCN*, copies/μL) and the number of target sequences per genome (*CNg*, copies/genome). Except for two of the *B*. *cereus* spores PrecipB extracts, where the average of duplicate qPCRs were used to determine the quantity values due to one of the triplicate reactions failing.

(3) $$\;cGE=\frac{qCN}{CNg}$$

The qPCR DNA concentration calculations were based on the organism’s genome mass (*GM*, ng/genome, Eq. 1) and the concentration of genome equivalents (*cGE*,GE/μL) (Eq. 4) [18,61].

(4) $$\;\left[DNA\right]=cGE\times GM$$

The three DNA concentration measurement methods were compared for each extraction method using a one-way ANOVA with Tukey’s HSD test for post hoc pair wise comparisons.

Extraction efficiency was calculated (Eq. 5) as the ratio of qPCR yield (*qY*, GE, Eq. 6) to the total number of cells extracted (*CE*, cells, Eq. 7), which was the product of the culture concentration (*CC*, cells/mL) and the volume of cells extracted (*VE*, mL).

(5) EE=qY/CE

(6) qY=cGE×EV

(7) CE=CC×VE

Replicate extraction efficiency outliers were detected using Grubb’s test, as implemented in R (outliers package) on extractions with more than two replicates. A series of one-way ANOVAs and post hoc pairwise comparison tests, Tukey’s HSD, were run in R on log transformed qPCR extraction efficiencies values for each extraction method. Extraction methods could not be compared due to unknown day-to-day variability in DNA extraction efficiency. ANOVA analysis was run on datasets without outliers.

### Availability of supporting data

The dataset supporting the results of this article is included as an additional file to the manuscript (Additional file 5).

## Competing interests

The author’s declare no financial or non-financial conflicts of interest.

## Authors’ contributions

NDO and JMB conceived of the study and its design, performed the data analysis, and wrote the manuscript. NDO conducted the experiments. Both authors read and approved the final manuscript.

## Supplementary Material

Additional file 1 **Table S1.** DNA A_260_/A_280_ and A_260_/A_230_ absorbance ratios. Click here for file

Additional file 2 **Figure S1.** Scatter Plot of A_260_/A_280_ and A_260_/A_230_ Ratios as a Function of a Control DNA (SRM 2372) Concentration. The experiments were executed on two separate days, A and B: with single (4 runs each) and triple (4 runs each) replicates respectively. The data point shape indicates day A, ●, B, ▲. The red vertical lines indicate the DNA concentration cutoff used for analysis of the DNA extract purity at 17.5 ng/μL. Click here for file

Additional file 3 **Figure S2.** Electropherograms for DNA Extracts Grouped by Cell Type and Extraction Method. The two manufacturer supplied markers are at 50 bp and 17000 bp. Dotted lines indicate the 1000 bp point on the x-axis. The bottom number within each graph is the number of replicates where the area under the curve for the full electropherogram was above the analysis threshold. The top two numbers are the mean and standard deviation for the percentage of DNA that was greater than 1000 bp (top value) and less than 300 bp (middle value). Scales are independent for each row due to the large range in responses. Click here for file

Additional file 4 **Figure S3.** Measured DNA concentration as a Function of the dilutions of the Human Quantification Standard (SRM 2372) concentration (ng/μL). Three dilution series replicates were processed, indicated in red, green, and blue, with five runs each. Dotted line indicates the defined limit of quantification (0.25 ng/μL) based on the increased observed variability for lower concentration dilutions. (PDF 94 kb) Click here for file

Additional file 5 Raw data (sheet labeled "DNAcompleteResults.csv") and metadata (sheet labeled "Head descriptions") used to support the results presented in this article. Click here for file
