# Supplementary material for: DNA extract characterization process for microbial detection methods development and validation
Source: BMC Res Notes. 2012 Dec 3;5:668. doi: 10.1186/1756-0500-5-668 (PMC3599793; doi:10.1186/1756-0500-5-668)
Supplement: Additional file 1 — Table S1. DNA A260/A280 and A260/A230 absorbance ratios. [file 1756-0500-5-668-S1.doc]

Supplemental Table 1. DNA A_260_/A_280_ and A_260_/A_230_ absorbance ratios.

| **Extraction Method** | **Cell Type** | **A_260_/A_280_^a^** | | | **A_260_/A_230_^a^** | | |
| --- | --- | --- | --- | --- | --- | --- | --- |
| **Reference** | *B. cereus* spores | 1.63^b^ | | | 1.16 | | |
|  | *S. cerevisiae* | 2.24 | ± | 0.00 | 2.38 | ± | 0.09 |
|  | *B. cereus* | 2.01 | ± | 0.02 | 1.67 | ± | 0.09 |
|  | *Burkholderia thailandensis* | 2.08 | ± | 0.01 | 2.19 | ± | 0.03 |
|  | *E. coli* | 2.06 | ± | 0.03 | 1.96 | ± | 0.07 |
| **PrecipB** | *S. cerevisiae* | 1.88 | | | 1.76 | | |
|  | *B. cereus* | 1.89 | ± | 0.02 | 1.68 | ± | 0.04 |
|  | *Burkholderia thailandensis* | 1.93 | ± | 0.04 | 1.72 | ± | 0.15 |
|  | *E. coli* | 1.89 | ± | 0.02 | 1.87 | ± | 0.12 |
| **ChemLysis** | *S. cerevisiae* | 2.05 | ± | 0.07 | 1.11 | ± | 0.13 |
|  | *Burkholderia thailandensis* | 2.17 | ± | 0.03 | 1.65 | ± | 0.09 |
|  | *E. coli* | 2.07 | ± | 0.01 | 1.91 | ± | 0.01 |
| **PrecipG** | *B. cereus* | 1.92 | ± | 0.04 | 1.77 | ± | 0.25 |
|  | *Burkholderia thailandensis* | 2.14 | ± | 0.17 | 1.16 | ± | 0.18 |
|  | *E. coli* | 1.91 | | | 2.02 | | |

^a^Ratio values were presented as mean ± standard deviation (N = 2 to 4). Samples with concentrations below the limit of quantification (17.5 ng/μL) were not included in the analysis.

^b^When one replicate extraction had a concentration above the limit of quantification the ratio for that replicate is shown.
