# Supplementary figures and images for: DNA extract characterization process for microbial detection methods development and validation
Source: BMC Res Notes. 2012 Dec 3;5:668. doi: 10.1186/1756-0500-5-668 (PMC3599793; doi:10.1186/1756-0500-5-668)

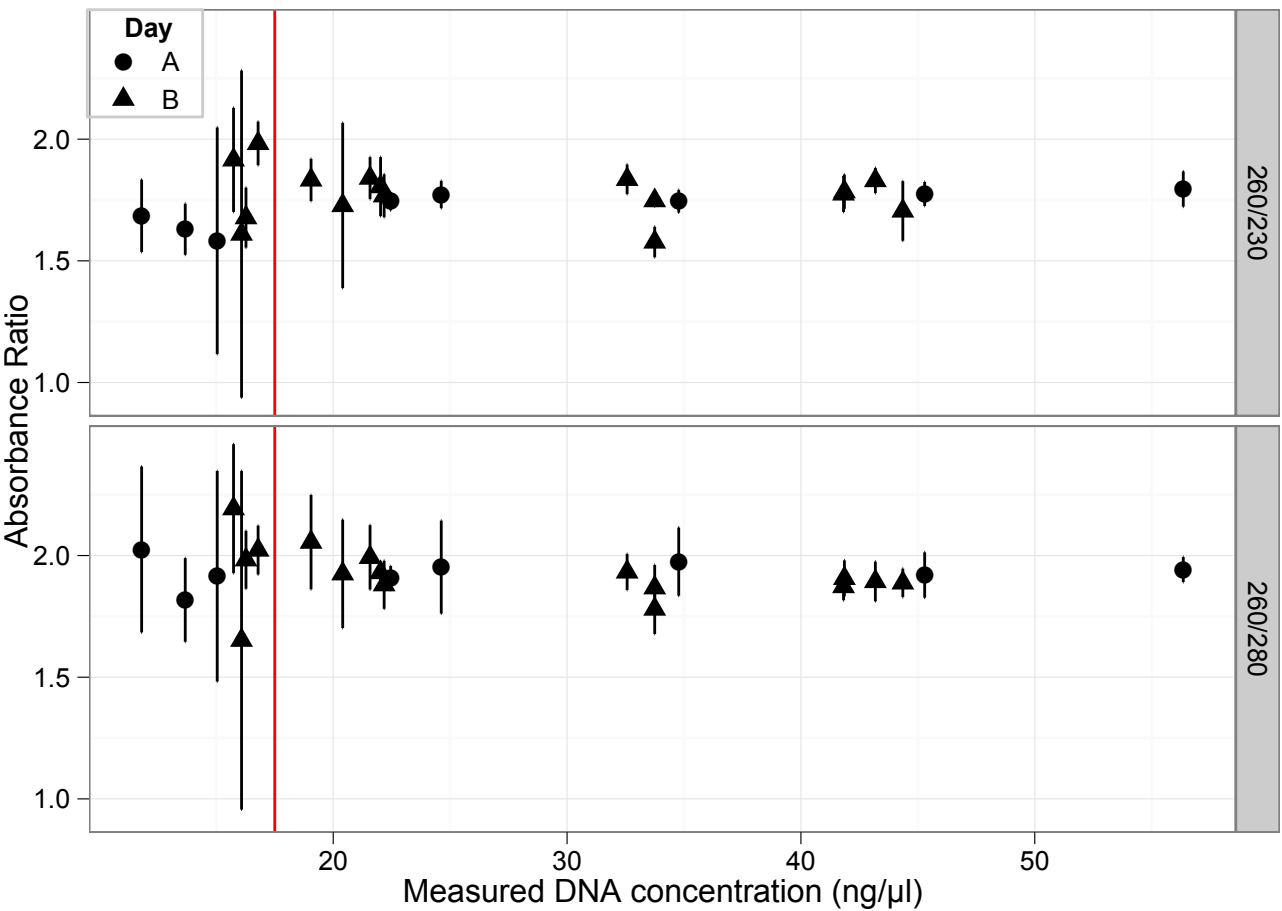

Supplement: Additional file 2 — Figure S1. Scatter Plot of A260/A280 and A260/A230 Ratios as a Function of a Control DNA (SRM 2372) Concentration. The experiments were executed on two separate days, A and B: with single (4 runs each) and triple (4 runs each) replicates respectively. The data point shape indicates day A, ●, B, ▲. The red vertical lines indicate the DNA concentration cutoff used for analysis of the DNA extract purity at 17.5 ng/μL. [file 1756-0500-5-668-S2.pdf]

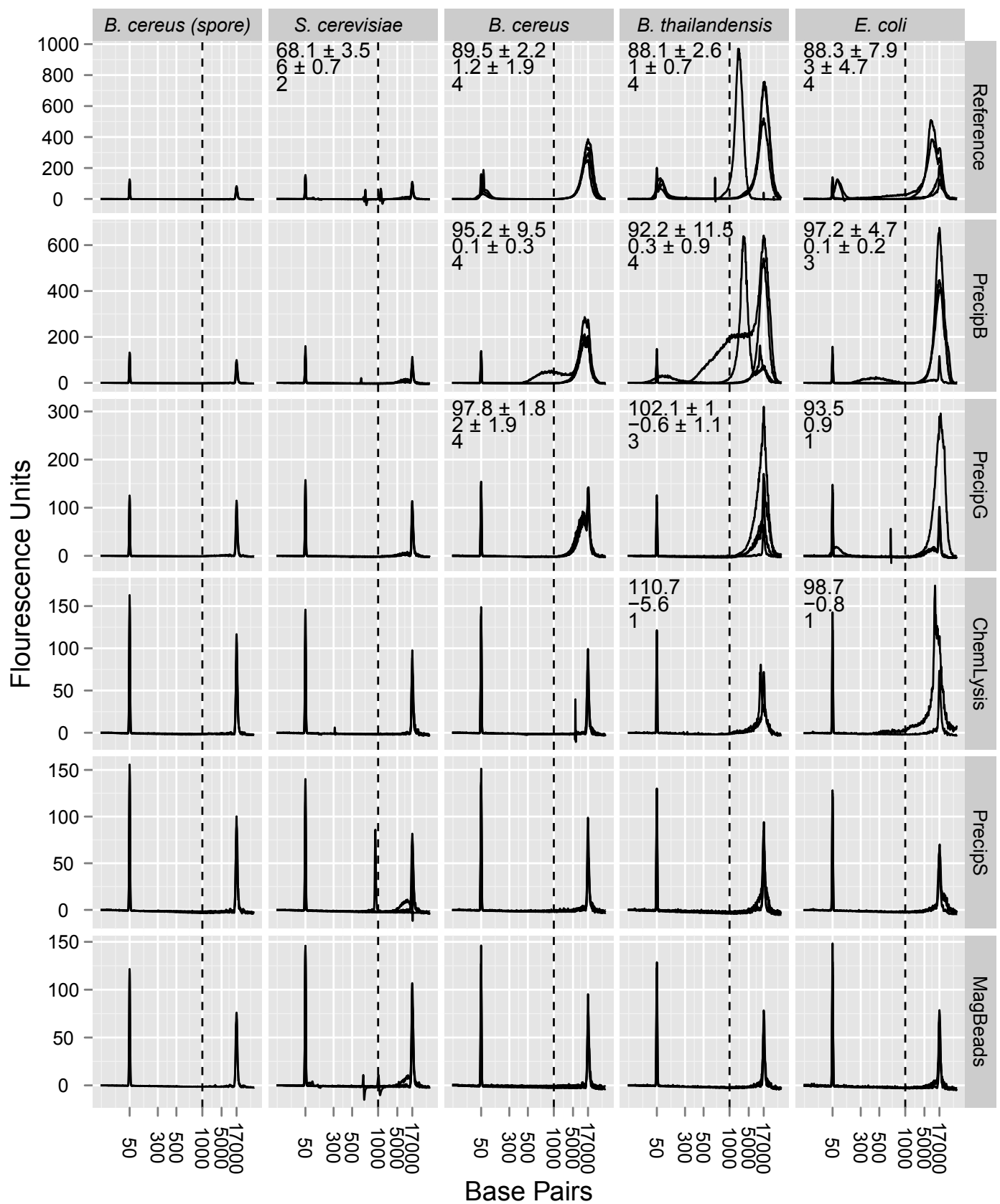

Supplement: Additional file 3 — Figure S2. Electropherograms for DNA Extracts Grouped by Cell Type and Extraction Method. The two manufacturer supplied markers are at 50 bp and 17000 bp. Dotted lines indicate the 1000 bp point on the x-axis. The bottom number within each graph is the number of replicates where the area under the curve for the full electropherogram was above the analysis threshold. The top two numbers are the mean and standard deviation for the percentage of DNA that was greater than 1000 bp (top value) and less than 300 bp (middle value). Scales are independent for each row due to the large range in responses. [file 1756-0500-5-668-S3.pdf]

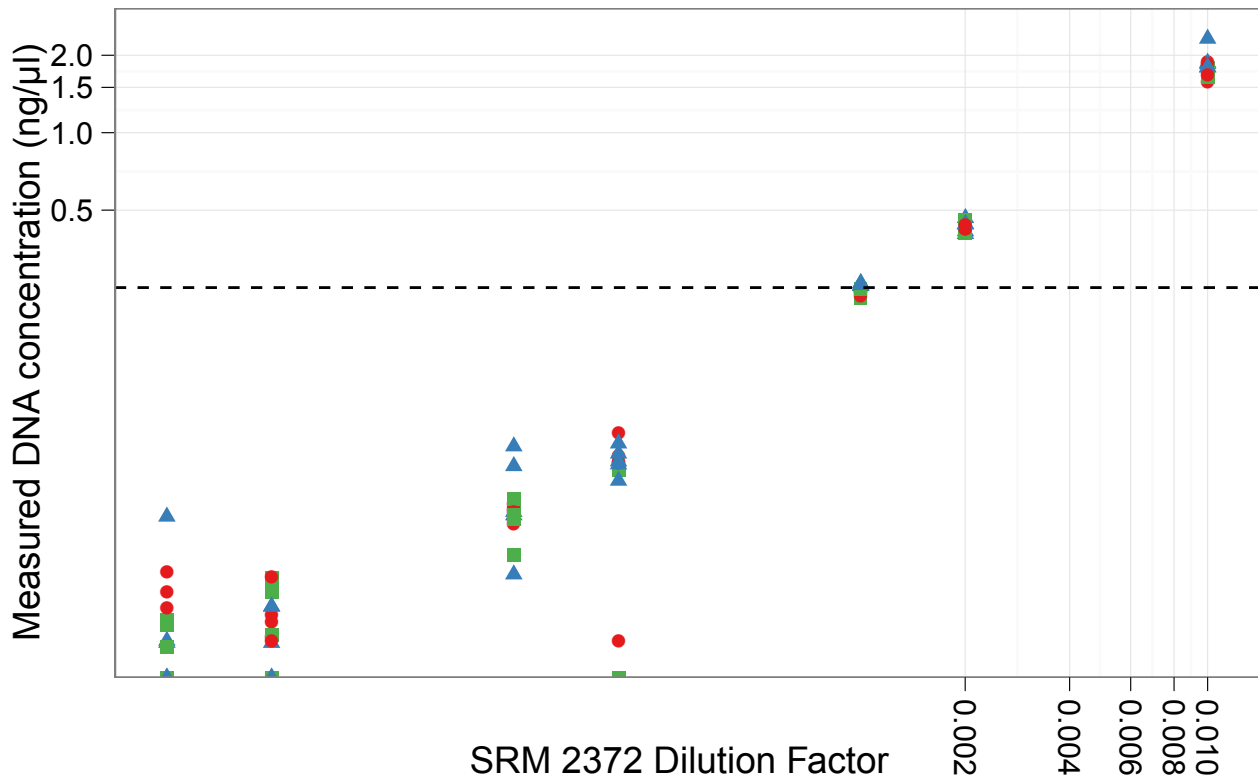

Supplement: Additional file 4 — Figure S3. Measured DNA concentration as a Function of the dilutions of the Human Quantification Standard (SRM 2372) concentration (ng/μL). Three dilution series replicates were processed, indicated in red, green, and blue, with five runs each. Dotted line indicates the defined limit of quantification (0.25 ng/μL) based on the increased observed variability for lower concentration dilutions. (PDF 94 kb) [file 1756-0500-5-668-S4.pdf]
